# Synthesis and Charaterization of Silica-Based Aldehyde Chitosan Hybrid Material for Biodiesel Purification

**DOI:** 10.3390/ma10101132

**Published:** 2017-09-25

**Authors:** Sandra Rodrigues da Silva, Nilson J. A. de Albuquerque, Rusiene M. de Almeida, Fabiane C. de Abreu

**Affiliations:** Instituto de Química e Biotecnologia, Universidade Federal de Alagoas (UFAL), Av. Lourival Melo Mota, s/n, Tabuleiro do Martins, Maceió-AL 57072-970, Brasil; ardnasrodrigues@yahoo.com.br (S.R.d.S.); nilson_braga1@hotmail.com (N.J.A.d.A.); rusiene.almeida@iqb.ufal.br (R.M.d.A.)

**Keywords:** modified chitosan, adsorbent, biodiesel

## Abstract

This study concerns the development and charaterization of Silica-based aldehyde Chitosan hybrid material as an adsorbent for biodiesel purification. This biocomposite was prepared by sol-gel route and oxidation with periodate, and then characterized. FTIR experiments showed that the hybrid formed presents absorption bands similar to those of Chitosan-Silica, with the exception of the vibrations at 1480 cm^−1^ and 1570 cm^−1^ attributed to the symmetrical angular deformation in the N-H plane, and possess large N_2_ Brunauer–Emmett–Teller (BET) surface areas. Thermogravimetric analysis (TG) and scanning electron microscopy (SEM) was also carried out. Adsorption studies of bioadsorbents involving the analysis of free glycerol, soap, acidity, diglycerides, triglycerides, and fluorescence spectroscopy showed that silica-based aldehyde chitosan has a good affinity for glycerol and a good purification process.

## 1. Introduction

There has been growing interest in recent years in the study and the development of materials in various areas (transport, housing, medicine, communications), as there is a need for improvement and more efficient use of already available resources.

The biocomposites developed an aim to interact with the environment in such a way that their impact is minimal during their useful life and that it is possible to recycle the materials of which they are composed or dispose of them in an ecologically friendly manner.

One highly attractive substance for studies in the field of biocomposites is chitosan, a natural polycationic biopolymer, which is usually synthesized from chitin [1,2]. The most important properties of chitosan include biocompatibility, biodegradability, and physical and chemical characteristics that enable polymer materials of different kinds (gels, flakes, fibers, pearls, microspheres, nanoparticles) to be obtained. These properties are useful in water treatment [3], cosmetics [4], the food industry [5], healthcare [6], agrochemicals [7], the production of paper [4], and textiles [8] and so forth. This biopolymer has been used as an adsorbent of various substances, such as metal ions, aromatic compounds, organic and inorganic acids, and colorings. This is due primarily to its structure, which contains hydrophilic and hydrophobic groups, as well as hydroxyls and amines, which confer powerful adsorptive properties [9,10,11].

Chitosan can be modified using various functional groups and/or inorganic compounds to increase the possibility of adsorption. These derivative compounds are generated by reactions of the hydroxyl and amine groups with appropriate reagents. One very attractive reagent for this end is tetraethylorthosilicate (TEOS), because of its compatibility with various biopolymers, including chitosan [12,13,14]. The composition of TEOS contains silanol groups (Si–OH) that easily bind to the hydroxyls, and amines present in chitosan, forming (chitosan/TEOS) composites with promising adsorptive properties [12,14], which can be exploited in various areas, including biodiesel purification. Another agent that is capable of modifying chitosan is the periodate ion. This reaction has been widely studied in various studies [15,16,17], in which the periodate ion acts selectively on the C–C 2,3 bond, generating dialdehydes, increasing the flexibility of the chain and giving rise to new reactive groups and new applications.

The aim of the present study was to obtain a composite based on chitosan, silica and various functional groups (amines, aldehydes, silanols) by modification with TEOS followed by partial oxidation with the periodate ion for adsorption studies. The biocomposite produced was used to remove free glycerol from biodiesel.

The biodiesel production process that is most commonly used in industry is transesterification, which requires a purification stage to ensure that the biofuel meets the necessary commercial specifications regarding contaminants, including free glycerol. According to Mazzieri [18], in high conversions (>97%), this is one of the main problems for obtaining pure biodiesel. The contaminant tends to polymerize in diesel engines, coating moving parts and causing corrosion and subsequent clogging of filters and fuel injection problems [19]. It may attract other contaminants, such as water, which increases the corrosiveness of biofuel and reduces the useful life of the engine, as well as producing noxious acrolein emissions [20]. Therefore, both ANP Resolution no 14/2012 and EN 14214 and ASTM D6751 specify a maximum limit for free glycerol in biodiesel of 0.02% m/m [21].

Adsorbents whose capacity to remove free glycerol from biodiesel has been evaluated include, magnesium silicate (Magnesol) [22,23], ion exchange resins (amberlite and pyrolite) [23], sulfonated resin [24], silicas [25], eucalyptus pulp [26], sugarcane bagasse [27], and cellulose [28]. However, researchers are still looking for a low-cost material that can be used on an industrial scale removes other impurities, and can be regenerated and reused. The adsorbents synthesized in the present study were characterized by FTIR, BET, TG and SEM. The kinetics of the adsorption process was investigated and glycerol adsorption isotherms produced for different temperatures. Other parameters, such as the alkalinity, acidity index, and fluorescence spectroscopy of purified biodiesel stored for three months were also investigated.

## 2. Results and Discussion

### 2.1. Synthesis

The first synthesis formed the chitosan-silica hybrid (CS). The interaction of chitosan with TEOS occurs by way of hydrogen bonds between amide and silanol groups, ionic bonds between the amine groups of chitosan and silanol groups, and covalent bonds derived from esterification of the hydroxide groups of chitosan with the silanol groups of silica [29].

**Scheme 1:**
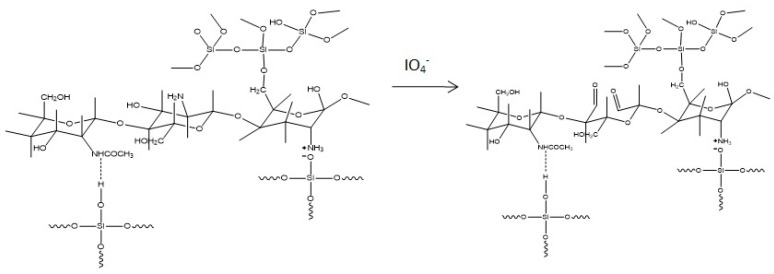
Schematic suggestion of CSP hybrid preparation.

According to Charhouf [17], dialdehyde is formed by cleavage of C2-C3 into GlcN units and these are capable of existing in equilibrium in the various forms (hydroxide, acyclical aldehydes, hemiacetals or hemialdals, or combinations of these). The suggested scheme for the reaction between CS and the periodate ion is presented in Scheme 1.

### 2.2. Characterization of CS and CSP

**Figure 1:**
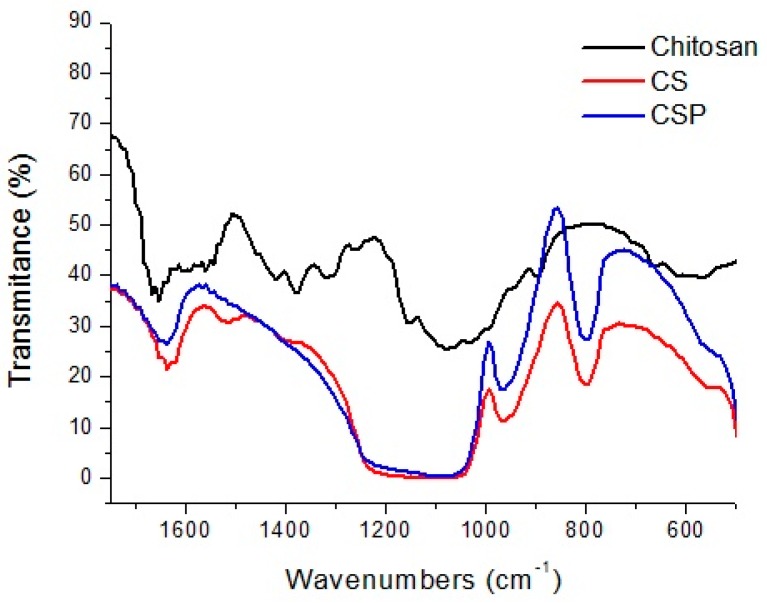
Infrared Spectra for CS and CSP adsorbents.

Fourier Transform Infrared (FTIR) spectroscopy analyses were conducted to observe the interaction of chitosan with TEOS and of the CS hybrid after reaction with periodate. The infrared spectra for chitosan, chitosan-silica, and CSP (Silica-Based Aldehyde Chitosan) up to 1600 and 4000 cm^−1^ are presented in Figure 1.

Chitosan presented the characteristic bands between 1654 cm^−1^ and 1080 cm^−1^, which correspond to the C=O stretching of the amide (1654 cm^−1^), N–H deformations of the primary amine (1570 cm^−1^), C–H vibrations of the acetamide group (1382 cm^−1^), and the saccharide structure of the polymer at 1080 cm^−1^.

The hybrid formed in the second synthesis (CSP) presents absorption bands similar to those of CS, with the exception of vibrations at 1480 cm^−1^ and 1570 cm^−1^ attributed to symmetrical angular deformation in the N–H plane.

The CSP spectrum was used to locate the band representing axial deformation of the carbonyl (C=O) of aldehyde, normally located at 1725 cm^−1^, but, according to Charhouf et al. [17] this is not easily characterized, since it can react with adjacent hydroxides to form intramolecular hemiacetals [16] and may also be covered by the band attributed to the angular deformation of the OH^−^ groups of water at 1650 cm^−1^.

The thermogravimetric (TG) curves and the first derivative (DTG) of the synthesized materials are presented below.

**Figure 2:**
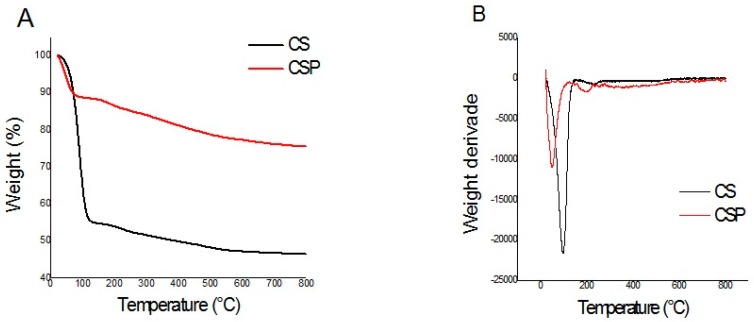
Thermogravimetric curves thermogravimetric analysis (TG) (**A**) and first derivative (DTG) (**B**) of adsorbent CS and CSP materials.

CS and CSP thermal behaviors were evaluated by TGA, as can be seen in Figure 2. For CS, the initial weight loss (45%) was observed around 140 °C and can be water (surface adsorbed) and products of the subsequent condensation of Si-OH groups. The second weight loss (3%) was observed at 260 °C due to the decomposition of low molecular weight species. A gradual loss was observed in the region of 265 °C up to 600 °C (around 5%). In this range of temperature probably occurred the dehydration of the saccharide rings, depolymerization and decomposition of the units of the organic polymer [29]. For CSP the initial weight loss (12%) is observed around 128 °C, a smaller loss as compared to CS. The second weight loss (3%) was observed at 227 °C. Then, the hybrid gradually lost 10% until the temperature of 800 °C. The higher temperature values for the first stage of decomposition observed for support when compared to the first stage decomposition temperature of the CS, indicates that the formation of the hybrid CSP was successful and this can be attributed to the formation of a more thermally stable composite.

Elemental analysis shows that the CSP adsorbents still contained carbonaceous material (Table 1).

**Figure 3:**
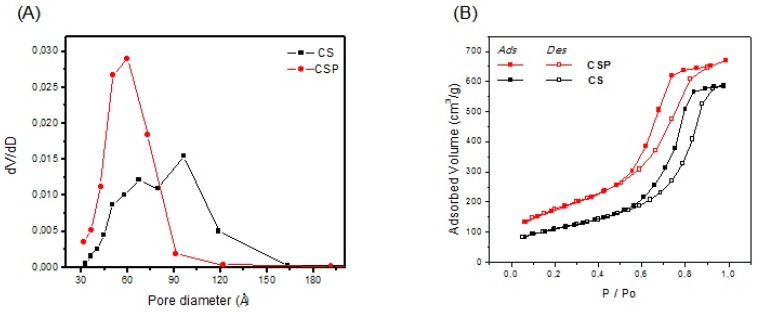
BET (**A**) and Nitrogen adsorption and desorption isotherm (**B**) for CS and CSP.

The results obtained for measurement of the surface area, mean pore diameter, pore volume, and the distribution of pore sizes for the synthesized materials (CS and CSP) are presented in Table 2 and Figure 3. The surface areas indicate that there was a significant increase when compared to that of chitosan (3.5 m^2^ g^−1^). Pore volume remained practically unchanged (Table 2).

The adsorbents should have a surface area and pores that enable unhindered access for all of the impurities to the internal surface to ensure the efficiency of the adsorption process.

Figure 3 shows the distribution of the pore size of the adsorbents synthesized in the present study. These adsorbents, CS and CSP, had pore diameters of 30 to 165 Å and 32 to 90 Å, respectively. According to Sing [30], these results classify the adsorbents as mesoporous materials (pore diameters of 20 to 200 Å). However, both the base substance (CS) and the material produced (CSP) have large specific surface areas and a pore structure adequate for use in biodiesel purification. According to Manuale et al. [25], this pore distribution enables the diffusion of impurities (glycerol, soap, mono-, di- and triglycerides) present in the biodiesel (Figure 3).

**Figure 4:**
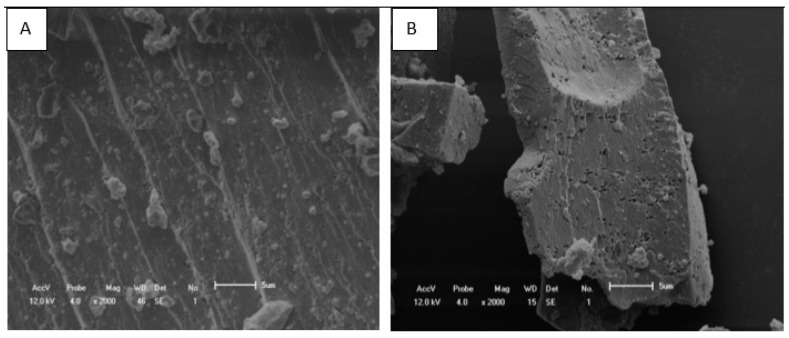
Scanning Electron Microscopy Images for CS (**A**) and CSP (**B**). The images are 5 micrometers with zoom of 2000×, respectively.

The morphology of the materials was studied by sweeping electron microscopy (SEM) and the results are presented in Figure 4.

Pure chitosan has an irregular surface with protuberances and small granules. After reaction with TEOS the surface showed a larger number of granules and ordered filaments. The CSP has a smoother porous surface.

### 2.3. Adsorption Studies

#### 2.3.1 Adsorption Isotherms of Free Glycerol for CS and CSP

**Figure 5:**
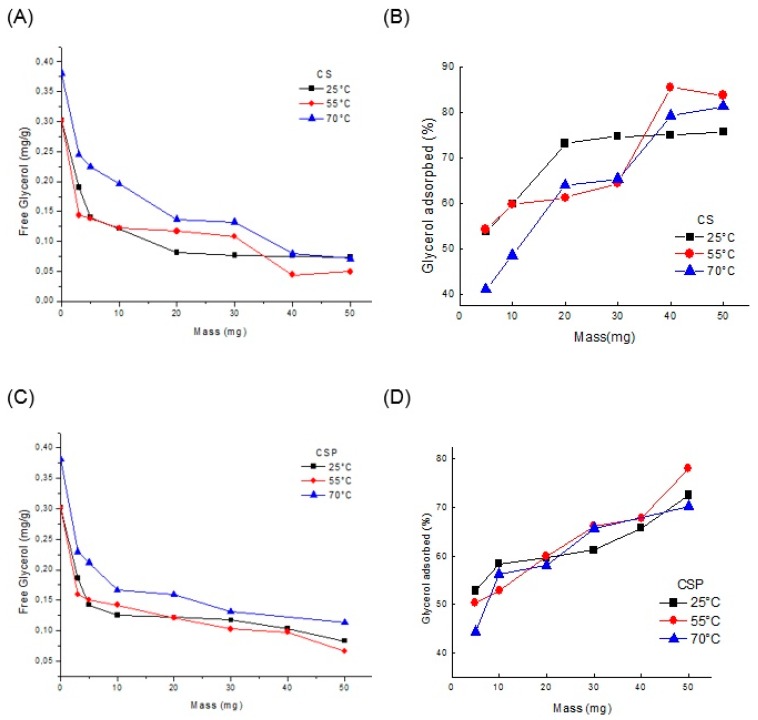
Influence of adsorbent loading on glycerine removal by CS (**A**,**B**) and CSP (**C**,**D**) for different temperatures.

The influence of adsorbent loading on the removal of glycerol from crude biodiesel by adsorbents is presented in Figure 5 for adsorption processes carried out at 25, 55, and 70 °C for 120 min. Crude biodiesel presented a glycerol content of 0.03 and 0.038 mg/g.

The CS adsorption process was improved by increasing the temperature from 25 °C to 55 °C, achieving 85% removal at a temperature of 55 °C. The maximum removal achieved at 25 °C was 74% (Figure 5B).

For the CSP, the maximum removal of 78% occurred at 55 °C and it was also found that this adsorbent could achieve higher percentages with an increased mass. Increasing the temperature from 55 to 70 °C did not improve the process for either adsorbent (Figure 5B,D).

However, the adsorbents were able to achieve the minimum concentration of free glycerol stipulated by the regulatory agencies (0.2 mg/g) with only 5 mg (in 5 mL of biodiesel) between the temperatures of 25 and 55 °C (Figure 5A,C). Gomes et al. [28] removed all glycerol using 1% (w/v) of cassava starch and rice starch at a temperature of 25 °C.

#### 2.3.2. Adsorption Kinetics of Free Glycerol for CS and CSP

**Figure 6:**
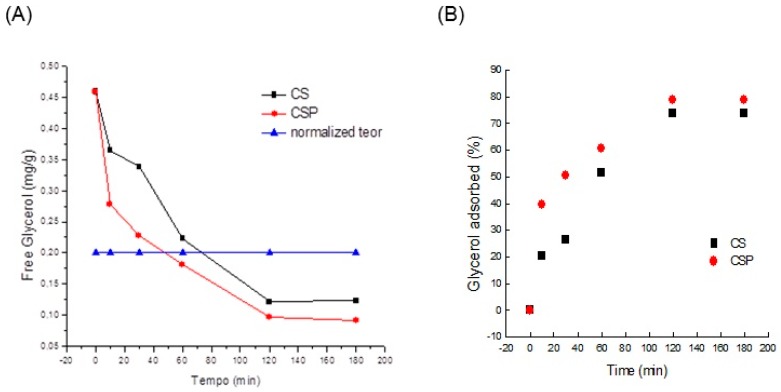
Influence of adsorption time on glycerine removal by CS and CSP. (**A**) Concentration of glycerol in biodiesel and (**B**) percentage of glycerol removed from biodiesel.

The adsorption kinetics of glycerol for the adsorbents (mass = 30 mg for 5 mL of biodiesel) was analyzed in terms of duration of contact at a temperature of 25 °C, as shown in Figure 6. Crude biodiesel presented a glycerol content of 0.46 mg/g.

The equilibrium point for achieving the maximum adsorption capacity of the adsorbents was obtained. The process of removal of glycerol from raw biodiesel using the adsorbents was efficient, taking only 60 min for CSP and 70 min for CS, thereby meeting the specification required by the regulatory agencies (0.2 mg/g) Figure 6A.

The kinetics of glycerol is rapid in the initial stage with around 50% adsorbed in a period of 1 h using CS and 60% using CSP (Figure 6B). The maximum removal of glycerol is achieved after 2 h, obtaining approximately 74% and 79% removal for CS and CSP, respectively, using 6% (w/v) of adsorbent. These percentages represent 0.122 and 0.092 mg/g (mg of glycerol/g in biodiesel), CS and CSP, which exceed the desired values (0.2 mg/g). These values show the high efficiency in the adsorption of glycerol.

These results are similar to those obtained by Alves et al. [27], who used 3.0 wt % of sugarcane bagasse and obtained an 80% removal.

### 2.4. Other Parameters: Alkalinity (Soap), Acidity, Diglycerides and Triglycerides

The biodiesel produced had combined an alkalinity (soap) of 1034.96 ppm and an acidity of 0.45 mg KOH/g. The purification process was shown to be efficient in terms of the removal of soap, with a reduction of more than 97% by using both CS and CSP. The American Society for Testing and Materials (ASTM D 6751) and the European Standard (EN 14214) limits this parameter to 200 ppm, although the quantity of sodium plus potassium is set at 5 ppm to ensure biodiesel quality. The quantity of NaOH obtained can be correlated to calculate the final quantities of sodium in biodiesel: 1.6 ppm (CS) and 2.2 ppm (CSP), with both adsorbents meeting the specifications. The established upper limit for acidity is 0.5 mg KOH/g and the acidity of the purified biodiesel produced lay within the acceptable limit (Table 3).

Chromatographic analysis obtained the removal of diglycerides and triglycerides from biodiesel with a high concentration of these contaminants. Qualitative determination was achieved by correlating the area of the peaks obtained with the concentration of the compound. The CS adsorbent performed best in removing diglycerides, adsorbing 80.4% as compared to 69.6% for CSP. CSP removed 78.4% of triglycerides and CS only 24.7% (Table 4).

**Figure 7:**
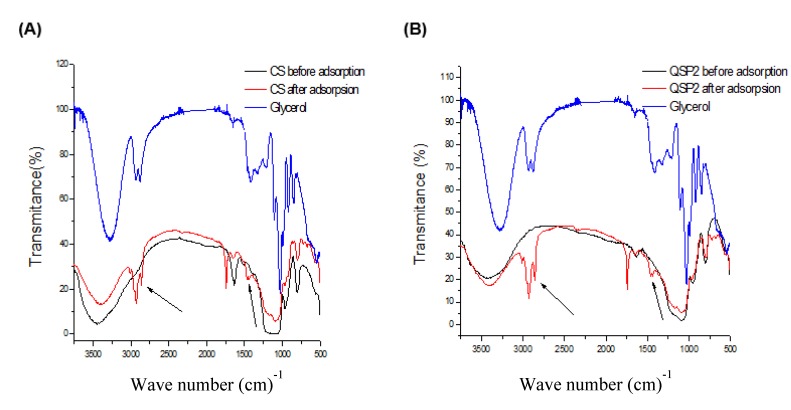
FTIR spectra of CS (**A**) and CSP (**B**) before and after adsorption.

After the adsorption study, the samples (biodiesel purified using CS and CSP) were stored in transparent receptacles and acidity and the fluorescence spectroscopy were measured after three months. FTIR analyses of the adsorbents were also conducted after adsorption, filtration and drying (Figure 7). Both adsorbents (CS and CSP) displayed glycerol peaks in the spectra.

An analysis of acidity showed that the samples purified using CS were four times more acidic, while the acidity of the sample purified using CSP remained practically unchanged. This shows that biodiesel purified by using CSP was more resistant to oxidation.

The antioxidant additives used to increase the stability of biodiesel are all characterized by chemical structures involve mono- and poly-hydroxyphenols with various substituents on the aromatic ring. The analysis of acidity thus suggests that the structure of CSP contains active groups (such as the hydroxyl group), which may provide prótons that inhibit the formation of free radicals and thus reduces the speed of oxidation.

Studies have shown [31,32] that this parameter can be used to evaluate stability with regard to oxidation and to monitor biodiesel quality during storage.

**Figure 8:**
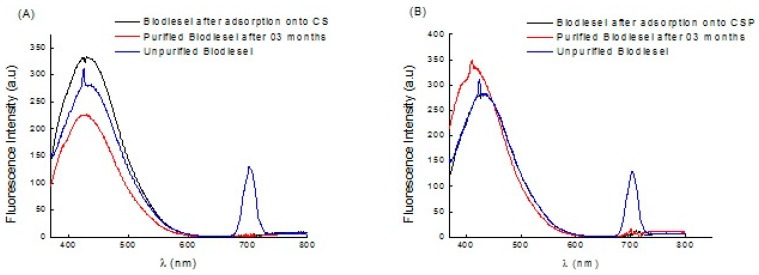
Fluorescence spectra for biodiesel alone and biodiesel after adsorption by (**A**) CS and (**B**) CSP.

Figure 8 shows the fluorescence spectra of samples of raw and stored biodiesel (after purification) diluted to 10^18^ molecules/cm^3^ in n-hexane.

It can be seen that all of the samples had a fluorescence of around 410 nm when excited at 350 nm, such as is characteristic of the compounds that make up biodiesel. The spectrum for raw biodiesel has one more region around 700 nm, characteristic of the chlorophyll in the soya oil used. This region did not appear after purification with either adsorbent.

The adsorbents react differently with biodiesel after purification and after storage. Biodiesel treated with CS has increased fluorescence but this diminishes after storage, owing to the formation of biodiesel oxidation compounds that reduce the transparency of the medium. The spectrum for biodiesel treated with CSP shows only the removal of chlorophyll, and, after storage, an increase in fluorescence in the region characteristic of esters. This behavior gives these adsorbents an advantage over others reported in the literature.

In fact, synthetic aldehyde chitosan hybrid material could be used for cleaning biodiesel through adsorption: binding the polar soap or glycerin molecules to its surfaces. This is probably an important mechanism for glycerin removal. Glycerin is removed from biodiesel through adsorption or filtration. Because glycerin is a highly polar molecule, it is held on the surface of the adsorbent by attraction to polar groups already on the surface. Soap, being soluble in glycerin, would be dissolved into the glycerin and removed from the biodiesel with it.

## 3. Materials and Methods

### 3.1. Materials

The raw materials employed to produce biodiesel, degummed soy oil, methanol (99.9%), and pure glycerol (>99.5%), were supplied by Sigma. Sodium stearate (99%) was chosen as the soap model and supplied by Serisa Química SRL. Glyceryl monostearate (98%) was used as the monoglyceride model and supplied by Cloretil SACIF. Silica samples were TriSyl 3000, 300B and 450 (provided by W.R. Grace Argentina SA). TEOS (99% purity) and periodate were purchased from Sigma-Aldrich (St. Louis, MO, USA) and used as received. Chitosan (deacetylation degree of 80%) was purchased from TCI-America (Portland, OR, USA).

### 3.2. Biodiesel Preparation

Biodiesel was prepared according to the standard procedure (alkaline transesterification via the methyl route, using NaOH as a catalyst), as described by Gomes et al. [28]. Many batches of the solution of the biodiesel product and unreacted methanol were synthesized and mixed together to provide a common biodiesel stock.

### 3.3. Synthesis of Chitosan-Silica Hybrid

Synthesis was carried out in accordance with Silva et al. [29], dissolving 0.250 g of chitosan in 10 mL of ethanol-water solution (1:4). The suspension was heated (60 °C) and agitated and 2 mL of concentrated hydrochloric acid was subsequently added. The solution was agitated for 12 h to achieve a full dissolution of the chitosan. After this, 10 mL of tetraethylorthosilicate was added and the solution was agitated for 40 min. The material produced was transferred to a glass plate and placed in a fume hood, where it was kept at ambient temperature for 12 h and solidified. It was then stored in a place protected from air and light.

### 3.4. Synthesis of CSP Compounds

One hundred mg of periodate was weighed out and transferred to a round-bottom flask. Fifty mL of acetic acid/sodium acetate buffer pH 4.5 and 1 g of CS were added. The flask was immersed in a bath at a temperature of 4 °C. The system was agitated for one hour and protected from light and nitrogen was added to de-aerate the medium.

The reaction was concluded by the addition of ethylenoglycol after one hour. The material produced was filtered in a vacuum and placed in a desiccator for 48 h. It was then stored in a place protected from air and light. This procedure was adapted from Vold and Christensen [15].

### 3.5. Characterization of Biosorbents

Textural characterization (BET surface area, pore volume, and pore size) of the silica and modified chitosan were measured by way of surface area and a porosity analyzer (Autosob I, Quantachrome Corporation, USA) using N_2_ adsorption at 77 K. Functional groups of the silica, chitosan, and silica based aldehyde chitosan (before and after the adsorption of IC) were detected using Fourier Transform Infrared (FTIR) spectroscopy. Before each analysis, the samples were dried at 60 °C under a vacuum for 72 h. The surface area characterizations of chitosan, chitosan silica, and chitosan silica aldehyde hybrid material were obtained.

The material produced was characterized by FTIR, thermogravimetric analysis (TGA), surface area determination (BET), scanning electron microscopy (SEM), and elemental analysis (Perkin Elmer).

For the Fourier Transform Infrared Spectrophotometer (Shimadzu, Prestige-21) experiments, the samples were prepared with KBr in the form of pellets. The region selected for analysis was medium infrared 4000–400cm^−1^ and the equipment was programmed to record an average of 20 sweeps.

Thermogravimetric analyses were carried out using a Shimadzu TGA-50, in nitrogen in a temperature band of 25 to 900 °C, at a heating velocity of 10 °C/min.

A CHNS-O Carlo Erba Instruments model EA 1110 elemental analyzer was used. The samples were weighed (2.3–2.7 mg) in an analytical scale in tin capsules. The procedure used helium gas flow and was carried out at a temperature of 1000 °C.

The adsorbents were activated in advance at 150 °C under a vacuum for 24 h and underwent liquid nitrogen adsorption and desorption using an automatic Quantachrome ChemBet-3000 Autosorb-1C physisorption instrument. The specific surface areas were calculated using the method described by Brunauer-Emmett-Teller, while the distributions of mean pore diameters were obtained using the Barret-Joyner-Halenda method.

A Shimadzu, SSX-550 Superscan model microscope and a Sanyu Electron, Quick Coater SC-701 model metalizer were used. The samples were metalized with gold, under a 10 mA current for 8 min.

### 3.6. Adsorption Studies

#### 3.6.1. Adsorption Isotherms

125 mL Erlenmeyer flasks were filled with 5 mL of biodiesel at different adsorption masses, preset at 3, 5, 10, 20, 30, 40, and 50 milligrams. The systems were placed in an incubator (shaker) and agitated (150 rpm) at a temperature of 27, 55, and 70 °C. The higher temperatures were selected as they are close to the temperature of biodiesel production [33]. After 120 min, the agitation was interrupted and aliquots of 50 µL were removed to measure free glycerol using UV/Vis analysis.

#### 3.6.2. Adsorption Kinetics

125 mL Erlenmeyer flasks were filled with 30 milligrams of adsorbent and 5 mL of biodiesel. The systems were placed in an incubator (shaker) and agitated at 150 rpm. After pre-set time periods (20, 40, 60, 120, and 180 min), agitation was interrupted and aliquots were removed to measure FG using UV/Vis analysis.

The free glycerol content was determined by UV/Vis using a calibration curve in a single phase system, following the method developed by Ribeiro and Rocha [34].

### 3.7. Other Parameters: Alkalinity, Acidity, Fluorescence, Diglycerides and Triglycerides

Biodiesel samples (5 mL) were purified with 100 mg of adsorbent under agitation for 2 h. Aliquots were collected for analysis of alkalinity, acidity, and fluorescence spectroscopy. The acidity and fluorescence analyses were repeated after three months.

Fluorescence spectroscopy measurements were obtained using an RF-5301PC (Shimadzu) spectrofluorometer. The readings were carried out by exciting the samples with 350 nm and monitoring emissions from 370 to 800 nm. The biodiesel samples were diluted in HPLC grade n-hexane at a concentration of 10^18^ molecules/cm^3^ (0.002 g/cm^3^).

Alkalinity was measured using the AOCS Cc17-95 method [35], and acidity by using EN 14104 [36].

For an analysis of diglycerides and triglycerides, biodiesel samples (10 mL) were purified with 100 mg of adsorbent under agitation for 2 h. High performance liquid chromatography (HPLC) was carried out using the method developed by Carvalho et al. [37].

## 4. Conclusions

Small quantities of the materials synthesized (CS and CSP) remove free glycerol from soya oil biodiesel, including low concentrations of the contaminant. These materials were also efficient in the removal of soap, diglycerides, and triglycerides. The adsorption isotherms show that the increase in temperature from 25 °C to 55 °C favors the adsorption process for both of the adsorbents. The most rapid removal kinetics was obtained with CSP, which, within one hour, had already achieved the concentration specified by regulatory agencies (0.02%). The adsorbents interact differently with soya biodiesel and the samples treated with CSP were more resistant to oxidation, as the adsorbent exhibited advantageous antioxidant properties.

However, the study showed the possibility of producing hybrid materials starting from sol-gel processes and periodate oxidation of chitosan that have promising adsorption applications.

## Figures and Tables

**Table 1:** Elemental analysis of carbon, Hydrogen and Nitrogen of CS and CSP adsorbents.

Adsorbent	Carbon (%)	Hydrogen (%)	Nitrogen (%)
CS	1.72	1.97	0.38
CSP	0.41	1.39	0.10

**Table 2:** Properties from Brunauer–Emmett–Teller (BET) analysis of CS and CSP materials.

Propriedades	QS	QSP
SBET (m^2^ g^−1^)	393.091	621.885
Diâmetro do poro (Å)	96.275	59.859
Volume do poros (cm)³/g	0.932	1.040

**Table 3:** Properties of Biodiesel after adsorption process.

Parameter	Raw Biodiesel	Purif CS	Purif QSP	Normal limits ^a^
Soap (ppm)	1034.96	21.8	29.03	200
Sodium (ppm)	78.3	1.6	2.2	5
Acidity (mg NaOH/g)	0.32	0.34	0.34	0.5

^a^ As specified by the ASTM D 6751 and EN 14214 quality standards.

**Table 4:** HPLC analysis of biodiesel before and after adsorption process.

Compound	Raw Bio (%)	CS (%)	CSP (%)
Diglycerides	9.2	1.8	2.799
Triglycerides	17	12.8	3.674
Esters	64	75.032	90.38
